# Correction: Efficacy and safety of prazequantel for the treatment of *Schistosoma mansoni* infection across different transmission settings in Amhara Regional State, northwest Ethiopia

**DOI:** 10.1371/journal.pone.0324187

**Published:** 2025-05-14

**Authors:** Getaneh Alemu, Arancha Amor, Endalkachew Nibret, Abaineh Munshea, Melaku Anegagrie

The word “praziquantel” is misspelled in the article title, as well as in the Abstract, Background, Tables 1, 3 and 4 caption, Results and Conclusion. In all of these cases, “prazequantel” should be “praziquantel”. The correct title is: Efficacy and safety of praziquantel for the treatment of *Schistosoma mansoni* infection across different transmission settings in Amhara Regional State, northwest Ethiopia. The correct citation is: Alemu G, Amor A, Nibret E, Munshea A, Anegagrie M (2024) Efficacy and safety of praziquantel for the treatment of *Schistosoma mansoni* infection across different transmission settings in Amhara Regional State, northwest Ethiopia. PLoS ONE 19(3): e0298332. https://doi.org/10.1371/journal.pone.0298332.

**Table 1:** Socio-demographic characteristics and STH co-infection among school-aged children participated in praziquantel efficacy testing in northwest Ethiopia, February to June 2023 (N = 110).

Variable	Catagory	Number	STH co-infection	
		(%)	Hookworms	*Ascaris lumbricoides*
			N(%)	N(%)
Age group (in years)	06-Sep	13(11.8)	0	0
	Oct-14	97(88.2)	11(11.3)	2(2.1)
Sex	Male	62(56.4)	7(11.3)	1(1.6)
	Female	48(43.6)	4(8.3)	1(2.1)
School Grade	01-Apr	72(65.5)	6(8.3)	2(2.8)
	05-Aug	38(34.5)	5(13.2)	0
District	Bahir Dar Zuria	17(15.5)	0	0
	Chagni town	34(30.9)	0	1(2.9)
	Dera	19(17.3)	10(52.6)	1(5.3)
	Tach Armachiho	40(36.4)	1(2.5)	0
School	Andasa Primary School	17(15.5)	0	0
	Chagni 01 Primary School	16(14.5)	0	0
	Chagni 03 Primary School	18(16.4)	0	1(5.6)
	Tach Qorata Primary School	19(17.3)	10(52.6)	1(5.3)
	Euket Amba Primary School	40(36.4)	1(2.5)	0

**Table 3:** Praziquantel cure rate and associated risk factors among school-aged children in northwest Ethiopia, February to June 2023 (N = 110).

Variable	Category	By KK				By POC-CCA (trace as negative)				By POC-CCA (trace as positive)			
		Cured(%)	Not cured (%)	*χ* ^2^	p-value	Cured (%)	Not cured(%)	χ^2^	p-value	Cured (%)	Not cured(%)	χ^2^	p-value
Age group	06-Sep	11(84.6)	2(15.4)	0.18	0.671	9(69.2)	4(30.8)	0.31	0.579	8(61.5)	5(38.5)	0.63	0.428
(in years)	Oct-14	86(88.7)	11(11.3)			74(76.3)	23(23.7)			70(72.2)	27(27.8)		
Sex	Male	54(87.1)	8(12.9)	0.16	0.689	45(72.6)	17(27.4)	0.63	0.426	42(67.7)	20(32.3)	0.69	0.406
	Female	43(89.6)	5(10.4)			38(79.2)	10(20.8)			36(75)	12(25)		
Grade level	01-Apr	65(90.3)	7(9.7)	0.88	0.349	57(79.2)	15(20.8)	1.55	0.213	52(72.2)	20(27.8)	0.17	0.676
	05-Aug	32(84.2)	6(15.8)			26(68.4)	12(31.6)			26(68.4)	12(31.6)		
District	Bahir Dar Zuria	16(94.1)	1(5.9)	2.14	0.544	14(82.4)	3(17.6)	0.54	0.91	14(82.4)	3(17.6)	2.58	0.461
	Chagni town	31(91.2)	3(8.8)			25(73.5)	9(26.5)			21(61.8)	13(38.2)		
	Dera	17(89.5)	2(10.5)			14(73.7)	5(26.3)			14(73.7)	5(26.3)		
	Tach Armachiho	33(82.5)	7(17.5)			30(75)	10(25)			29(72.5)	11(27.5)		
School	Andasa	16(94.1)	1(5.9)	2.53	0.639	14(82.4)	3(17.6)	2.53	0.64	14(82.4)	3(17.6)	4.61	0.33
	Chagni 01	14(87.5)	2(12.5)			10(62.5)	6(37.5)			8(50)	8(50)		
	Chagni 03	17(94.4)	1(5.6)			15(83.3)	3(16.7)			13(72.2)	5(27.8)		
	Tach Qorata	17(89.5)	2(10.5)			14(73.7)	5(26.3)			14(73.7)	5(26.3)		
	Euket Amba	33(82.5)	7(17.5)			30(75)	10(25)			29(72.5)	11(27.5)		
SCH endemicity^*^	Low	33 (91.7)	3 (8.3)	1.95	0.377	28(77.8)	8(22.2)	0.18	0.915	28(77.8)	8(22.2)	2.25	0.325
	Moderate	31(91.2)	3 (8.8)			25(73.5)	9(26.5)			21(61.8)	13(38.2)		
	High	33(82.5)	7 (17.5)			30(75)	10(25)			29(72.5)	11(27.5)		
Intensity of infection	Light	66(95.7)	3(4.3)	12.53	0.002	55(79.7)	14(20.3)	3.24	0.198	54(78.3)	15(21.7)	4.87	0.088
	Moderate	22(81.5)	5(18.5)			20(74.1)	7(25.9)			16(59.3)	11(40.7)		
	Heavy	9(64.3)	5(35.7)			8(57.1)	6(42.9)			8(57.1)	6(42.9)		
STH co-infection	Yes	12(92.3)	1(7.7)	0.24	0.624	10(76.9)	3(23.1)	0.02	0.896	10(76.9)	3(23.1)	0.26	0.611
	No	85(87.6)	12(12.4)			73(75.3)	24(24.5)			68(70.1)	29(29.9)		
Overall		97(88.2)	13(11.8)			83(75.5)	27(24.5)			78(70.9)	32(29.1)		

**Table 4:** Adverse events observed among school-aged children after praziquantel treatment across associated risk factors in northwest Ethiopia, February to June 2023 (N = 110).

Variable	Category	Number of children assessed	Adverse event	χ^2^	P-value
		n	n(%)		
Age group	06-Sep	13	6(46.2)	3.72	0.054
(in years)	Oct-14	97	21(21.6)		
Sex	Male	62	11(17.7)	3.55	0.06
	Female	48	16(33.3)		
Transmission setting of data collection sites	Low	36	6(16.7)	2.59	0.274
	Moderate	34	8(23.5)		
	Heavy	40	13(32.5)		
Intensity of infection	Light	69	14(20.3)	5.29	0.071
	Moderate	27	11(40.7)		
	Heavy	14	2(14.3))		
STH co-infection	Yes	13	0(0.0)	4.8	0.029
	No	97	27(27.8)		
Number of PZQ tablets taken	<3 tablets	96	23(24.0)	0.14	0.743
	≥3 tablets	14	4(28.6)		
Overall		110	27(24.5)		

The correct captions for Tables 1, 3 and 4 have been provided here:

In the Results subsection of the Abstract, there is an error in the eighth sentence of the paragraph. The correct sentence is: Eleven (10.0%), eight (7.3%), six (5.5%), and two (1.8%) participants complained about abdominal pain, nausea, headache, and anorexia, respectively.

In the Adverse events after prazequantel treatment subsection of the Results, there is an error in the second sentence of the paragraph. The correct sentence is: The adverse events reported were abdominal pain (11/110, 10.0%), nausea (8/110, 7.3%), headache (6/110, 5.5%), and anorexia (2/110, 1.8%).

In the Discussion section, there is an error in the fourth sentence of the first paragraph. The correct sentence is: The CR in the present study was higher than previous reports from Ethiopia [20,43] and other SSA countries [44–46].

There is an error in reference 21. The correct reference is: Tesfie A, Getnet G, Abere A, Yihenew G, Belete Y, Kassa M, Gize A. Praziquantel is an effective drug for the treatment of Schistosoma Mansoni infection among school-aged children in Northwest Ethiopia. Trop Med Health. 2020; 48:28. doi: 10.1186/s41182-020-00204-z.
