# Supplementary material for: Efficacy and safety of prazequantel for the treatment of Schistosoma mansoni infection across different transmission settings in Amhara Regional State, northwest Ethiopia
Source: PLoS One. 2024 Mar 4;19(3):e0298332. doi: 10.1371/journal.pone.0298332 (PMC10911589; doi:10.1371/journal.pone.0298332)
Supplement: S1 File — (DOCX) [file pone.0298332.s001.docx]

|  | | **Baseline EPG** | | | | | | **Follow up EPG** | | | | | |
| --- | --- | --- | --- | --- | --- | --- | --- | --- | --- | --- | --- | --- | --- |
| **Variable** | **Category** | **mean + SD** | **Median** | **Mode** | **Minimum** | **Maximum** | **Range** | **mean + SD** | **Median** | **Mode** | **Minimum** | **Maximum** | **Range** |
| Age group  (in years) | 6-9 | 116.3 + 95.4 | 72.0 | 48.0^a^ | 24.0 | 360.0 | 336 | 11.1 + 28.8 | 0 | 0 | 0 | 96.0 | 96.0 |
|  | 10-14 | 170.2 + 215.4 | 72.0 | 24.0 | 12.0 | 1104.0 | 1092.0 | 6.7 + 23.2 | 0 | 0 | 0 | 168.0 | 168.0 |
| Sex | Male | 180.0 + 235.6 | 48.0 | 24.0 | 24.0 | 1104.0 | 1080.0 | 6.2 + 18.4 | 0 | 0 | 0 | 96.0 | 96.0 |
|  | Female | 143.0 + 158.0 | 72.0 | 48.0 | 12.0 | 648.0 | 636.0 | 8.5 + 29.5 | 0 | 0 | 0 | 168.0 | 168.0 |
| Intensity of infection | Light | 47.3 + 23.3 | 48.0 | 24.0 | 12.0 | 96.0 | 84.0 | 3.1 + 20.5 | 0 | 0 | 0 | 96.0 | 96.0 |
|  | Moderate | 228.4 + 81.6 | 240.0 | 120.0 | 120.0 | 384.0 | 264.0 | 10.7 + 24.3 | 0 | 0 | 0 | 168.0 | 168.0 |
|  | Heavy | 613.7 + 196.7 | 576.0 | 432.0 | 408.0 | 1104.0 | 696.0 | 20.6 + 32.4 | 0 | - | - | - | - |
| STH co-infection | Yes | 42.5 + 24.3 | 24.0 | 24.0 | 24.0 | 96.0 | 72.0 | 1.9 + 6.7 | 0 | 0 | 0 | 24.0 | 24.0 |
|  | No | 180.1 + 213.4 | 72.0 | 48.0 | 12.0 | 1104.0 | 1092.0 | 7.9 + 25.2 | 0 | 0 | 0 | 168.0 | 168.0 |
| Overall |  | 163.9 + 205.3 | 72.0 | 24.0 | 12.0 | 1104.0 | 1092.0 | 7.2 + 23.8 | 0 | 0 | 0 | 168.0 | 168.0 |
| Overall fecal egg counts at follow-up calculated only from non-cured participants (n=13) | | | | | | | | 60.9 **+** 39.9 | 48.0 | 48.0 | 24.0 | 168.0 | 144.0 |

1. Mean, median, mode, minimum, maximum and range of *S. mansoni* fecal egg counts among schoolchildren in northwest Ethiopia, February to June 2023 (N = 110)

^a^ two modes (48.0 and 72.0) exist each reported 3 times.

1. Distribution of light, moderate and heavy intensity infections by transmission setting (N = 110)

|  | | Transmission setting | | | Total | χ^2^, *p-value* |
| --- | --- | --- | --- | --- | --- | --- |
|  |  | Low | Moderate | High |  |  |
| Infection intensity | Light | 32 | 22 | 15 | 69 | 24.88  <0.001 |
|  | Moderate | 3 | 10 | 14 | 27 |  |
|  | Heavy | 1 | 2 | 11 | 14 |  |
| Total | | 36 | 34 | 40 | 110 |  |
