# Supplementary material for: Efficacy and safety of prazequantel for the treatment of Schistosoma mansoni infection across different transmission settings in Amhara Regional State, northwest Ethiopia
Source: PLoS One. 2024 Mar 4;19(3):e0298332. doi: 10.1371/journal.pone.0298332 (PMC10911589; doi:10.1371/journal.pone.0298332)
Supplement: S2 File — (DOCX) [file pone.0298332.s002.docx]

Mean fecal egg count and egg reduction rate among schoolchildren in northwest Ethiopia, February to June 2023 (N = 110).

| **Variable** | **Category** | **Baseline EPG (mean + SD)** | **P-value** | **Follow up EPG (mean + SD)** | **P-value** | **ERR**  **% (95%CI)** |
| --- | --- | --- | --- | --- | --- | --- |
| Age group  (in years) | 6-9  10-14 | 116.3 + 95.4  170.2 + 215.4 | 0.637 | 11.1 + 28.8  6.7 + 23.2 | 0.626 | 94.9(87.4-100.0)  93.3(84.7-99.0) |
| Sex | Male  Female | 180.0 + 235.6  143.0 + 158.0 | 0.507 | 6.2 + 18.4  8.5 + 29.5 | 0.763 | 95.7(90.5-99.4)  90.7(74.0-99.6) |
| Intensity of infection | Light  Moderate  Heavy | 47.3 + 23.3  228.4 + 81.6  613.7 + 196.7 | <0.001 | 3.1 + 20.5  10.7 + 24.3  20.6 + 32.4 | 0.002 | 92.0(79.2-100.0)  95.8(91.5-99.2)  96.6(93.5-98.9) |
| STH co-infection | Yes  No | 42.5 + 24.3  180.1 + 213.4 | 0.002 | 1.9 + 6.7  7.9 + 25.2 | 0.569 | 92.3(75.0-100.0)  93.7(85.2-98.9) |
| Overall |  | 163.9 + 205.3 |  | 7.2 + 23.8 |  | 93.5(85.4-98.5) |
