# Supplementary material for: Efficacy and safety of prazequantel for the treatment of Schistosoma mansoni infection across different transmission settings in Amhara Regional State, northwest Ethiopia
Source: PLoS One. 2024 Mar 4;19(3):e0298332. doi: 10.1371/journal.pone.0298332 (PMC10911589; doi:10.1371/journal.pone.0298332)
Supplement: Attachment — Submitted filename: Responses to reviewers-2.docx [file pone.0298332.s005.docx]

**Responses to Reviewers’ Comments and Questions**

Dear editor and reviewers, thank you for your constructive comments again which all are important inputs for the betterment of the manuscript. Below we tried to respond to all the comments/questions one by one; we also have incorporated all the corrections in the revised manuscript (shown as highlighted).

**Editor**

Please review your reference list to ensure that it is complete and correct. If you have cited papers that have been retracted, please include the rationale for doing so in the manuscript text, or remove these references and replace them with relevant current references.

**Response**: we have re-checked all the references and we have confirmed all references in the ‘references’ list are correctly cited in the body of the manuscript; and all cited references are included in the list. We have also ensured that the references and citations are made based on the journal guideline. We did not cite any paper that has been retracted.

**Reviewer #1**: 
Abstract: nothing mentioned about efficacy/safety in different transmission settings. Since this is something that makes this manuscript unique, I think it would be good to give it some attention in the abstract.

**Response**: comment accepted and we have added results about CR and adverse events across the three transmission settings (highlighted). We have also added data about adverse events in different transmission settings in Table 4.
Line 114-116: here ‘in different transmission settings’ should be added to the objective of the study. ‘Hence, the present study aimed to assess PZQ efficacy and safety for the treatment of *S. mansoni* infection and to compare the capacity of POC-CCA and KK for assessing cure in different transmission settings’.
**Response**: we accept the comment and have amended the objective accordingly.
Sample size calculation:
Prevalences are based on microscopy I believe, this should be added to the text to be clear since you will be comparing microscopy with POC-CCA.

**Response:** we accept the comment and we have explained this in the revised manuscript.
I do not understand the 29% prevalence in low endemic area, how can this be so ‘high’? I think the authors are trying to explain this, but it remains unclear to me.
**Response:** we expect it will be confusing for readers (that is why we tried to explain in the manuscript). In Ethiopia, Schistosomiasis mapping was done at a district level. They took samples from schools a district and the endemicity level of the district was determined based on the mean prevalence from schools. However, there might be great variations across schools or villages within the same district. So, if researchers recruit participants from a school/village where there is high *Schistosoma* focal transmission, the prevalence might be higher than the respective district’s average prevalence.

I would suggest to make a new paragraph of ‘Sampling technique’
**Response**: comment accepted and we have revised the manuscript accordingly
Table 4. Number of children assessed, the percentage should be of the subgroup, not of the total group. For example, now it looks like only 12% of the 6-9 year olds were examined for adverse events, while all of them were actually examined. Unless in some subgroups not all children were assessed, I do not see a reason why this column should be added to the table.

**Response**: it was just to give information about the proportion of each group among the total. Now we ensured that such information is presented in Table 1 and we have removed the percentage in Table 4, to avoid confusions.
What was the distribution of low/moderate/heavy intensity infections among the 3 endemicity districts? I know the numbers might be small, but I would be interested to see this. For example, do all the heavy intensity infections come from the high endemic area? Or do you also find them in the low endemic area? It would be nice to add this information to the first table, or as supplementary material or even added in a few lines in the text. I think this is very informative.

**Response:** Thank you. We have presented this data as supporting information (second table in Supporting information S1) in the revised submission. We have also explained this in the ‘results’ section.

**Reviewer #2:**

This study has made appropriate corrections during the review process, therefore, I suggest that it be accepted.

Thank you
