# Supplementary material for: Efficacy and safety of prazequantel for the treatment of Schistosoma mansoni infection across different transmission settings in Amhara Regional State, northwest Ethiopia
Source: PLoS One. 2024 Mar 4;19(3):e0298332. doi: 10.1371/journal.pone.0298332 (PMC10911589; doi:10.1371/journal.pone.0298332)
Supplement: Attachment — Submitted filename: Reviewer Comment (6 12 2023).docx [file pone.0298332.s003.docx]

**Author   :** Tukwarlba et *al*.

**Title       :** Efficacy and safety of praziquantel for the treatment of Schistosoma mansoni infection

across different transmission settings in Amhara Regional State, northwest Ethiopia

**Journal    :** PLoS Negl Trop Dis

**Reference:** PONE-D-23-33684

**General comment**

The authors of the current study are working on a well-known issue. The data could be useful for Ethiopia. Here I have some comments and suggestion for improvement.

**Title**

= “**Efficacy and safety of praziquantel for the treatment of *Schistosoma mansoni* infection across different transmission settings in Amhara Regional State, northwest Ethiopia**” This title should be revised to consider the content of the MS. Note sure if this study was implemented in several transmission settings. What is clear is that the study was targeting SAC with different level of infection intensity (low, moderate and heavy)

**Abstract**

= “Factors such as reduced PZQ efficacy after … contributing to persistent transmission of the disease.” Regarding the context in Ethiopia, this could not the rationale behind this study. The authors should find good rationale supporting the current work

**Introduction**

= “More than 240 million people are infected globally” This statement is not anymore true

= “Reduced PZQ efficacy following multiple rounds of MDA might have contributed for the continued incidence and this has been evidenced by recent studies.” As mention above the rationale behind this study must be reconsidered.

**Materials and methods**

= “From February to June 2023, a school-based single-arm ….. different transmission settings of intestinal schistosomiasis” Which are these different settings? How those settings have been selected?

= “the calculated sample size was 635. For the PZQ efficacy study, we selected the above mentioned five schools to which a total of 283 participants were allocated.” What is the precise sample size of this work? How precisely this sample size has been estimated? Please, generate a new section entitled “Sample size calculation” and describe clearly how the sample size has been assessed.

= Please, define clearly your primary outcome and secondary outcome and show us how this has been considered in your sample size calculation.

= L151-L161 “**Urine POC-CCA test**” this part could be shorten avoiding speculation

= “Duplicate KK slides were prepared and examined from each sample and the average egg count was taken as the final EPG” This statement is not correct. Please, revise since 24 to get the EPG should multiply the average egg count from both slides.

=”Schoolchildren who lived in the study area at least for six months, attended a regular primary school, gave consent/assent to participate in the study were included.” SAC could not consent they are only assenting and their parents are consenting.

=”**Evaluation of praziquantel efficacy**” Please specify the brand of the praziquantel used in the current study

= L188 – L190 “Treated children were then counseled to avoid activities related to *S. mansoni* infection …. in a pot/jar for ≥24 hours before use).” Remove this part since praziquantel is not active against juvenile worms. So, whatever the precautions you are taking to avoid reinfection you could not clear the juvenile work in the children body. The follow up time set to three weeks has been set to limit the impact of reinfection on cure rate and ERR calculation.

= How many time the adverse events have been follow ?

= L214: “STH infected vs non-infected” This is the first time STH are mentioned. Nowhere, in the previous section something is said about STH.

= L214: “while Kruskal-Wallis H test was used to compare the mean EPG among participants with light, moderate and heavy intensity of infections” Why this comparison since the groups are already well defined as low, moderate and heavy infected participants?

= L234: Please write “**Cure rate**” instead of “**Cure rate and associated risk factors**”

**Results**

**=** Figure 1: should be revised to make it understandable including a figure caption

**=** Table 4: Please, could you provide confidence interval for the ERR

= Table 5 should be revised as follow:

| **Variable** | **Category** | **Number of children assessed** | **Adverse event**  **n (%)** | **χ2** | **P-value** |
| --- | --- | --- | --- | --- | --- |
|  |  |  |  |  |  |
|  |  |  |  |  |  |

**Discussion**

= “The CR among SC with light infection (95.7%) by KK was higher than the CR among SC with moderate (81.5%) and heavy (64.3%) infections (χ2 = 12.53, p = 0.002)” One issue here is also the insensitivity of the KK in low infected people and need to be discussed.

= Please, make you discussion around your key findings by avoiding speculation

= Due to the limited sample size in the different study groups, the results of the current study should be interpreted with caution.
